# MultiplexSSR: A pipeline for developing multiplex SSR‐PCR assays from resequencing data

**DOI:** 10.1002/ece3.6121

**Published:** 2020-03-04

**Authors:** Liang Guo, Quan Yang, Jing‐Wen Yang, Nan Zhang, Bao‐Suo Liu, Ke‐Cheng Zhu, Hua‐Yang Guo, Shi‐Gui Jiang, Dian‐Chang Zhang

**Affiliations:** ^1^ Key Laboratory of South China Sea Fishery Resources Exploitation and Utilization Ministry of Agriculture and Rural Affairs South China Sea Fisheries Research Institute Chinese Academy of Fishery Sciences Guangzhou China; ^2^ Guangdong Provincial Engineer Technology Research Center of Marine Biological Seed Industry Guangzhou China; ^3^ National Demonstration Center for Experimental Fisheries Science Education Shanghai Ocean University Shanghai China

**Keywords:** multiplex SSR‐PCR, pedigree construction, pipeline, resequencing

## Abstract

Next‐generation sequencing has greatly promoted the investigation of single nucleotide polymorphisms, while studies of simple sequence repeats are sharply decreasing. However, simple sequence repeats still present some advantages in conservation genetics. In this study, an end‐to‐end pipeline referred to as MultiplexSSR was established to develop multiplex PCR assays in batches with highly polymorphic simple sequence repeats for capillary platforms from resequencing data. The distribution of single sequence repeats in the genome, the error profiles of genotypes and allelotypes, and the increase in the allele length range depending on the number of individuals were investigated. A total of 98% of single sequence repeats presented lengths of less than 100 bp. The error rate of the genotyping and allelotyping of dimeric patterns was ten times higher than those for other patterns. The error rate of allelotyping was less than that of genotyping. The allele length range reached approximate saturation with 10 individuals. This pipeline uses allele numbers to select highly polymorphic loci, masks loci with variation, and applies in silico PCR to improve primer specificity. The application of the developed multiplex SSR‐PCR assays validated the pipeline's robustness, showing higher polymorphism and stability for the developed simple sequence repeats and a lower cost for genotyping and providing low‐depth resequencing data from less than a dozen individuals for the development of markers. This pipeline fills the gap between next‐generation sequencing and multiplex SSR‐PCR.

## Graphical abstract

We established a pipeline referred to as MultiplexSSR to develop mutiplex SSR‐PCR from population resequencing data. The application of developed multiplex SSR‐PCRs validated this pipeline's robustness, showing higher polymorphism and stability for the developed simple sequence repeats and a lower cost for genotyping and providing low‐depth resequencing data from less than a dozen individuals for the development of markers.

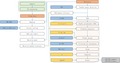

## 1 INTRODUCTION

Since the 1990s, simple sequence repeat (SSR) markers have played a major role in the fields of ecology and genetics. However, with the release of next‐generation sequence platforms, single nucleotide polymorphism (SNP) markers have increasingly been used, while the use of SSRs has decreased because of the difficulty of their development and genotyping (Ashton, Ritchie, & Wellenreuther, 2017; Flanagan & Jones, 2019; Hodel et al., 2016; Vieira, Santini, Diniz, & Munhoz, 2016). Commonly employed algorithms usually balance speed and accuracy and always focus on SNPs. There are also algorithms that focus on SSRs, such as lobSTR (Gymrek, Golan, Rosset, & Erlich, 2012) and RepeatSeq (Highnam et al., 2013). However, the detected SSRs generally serve as a supplement to SNPs rather than being independently applied. Compared with SNPs, the detection of SSRs is much more costly and requires especially high coverage because of the errors caused by stutter, requiring reads to span the repeat region, and because these markers are distributed at a low density throughout the genome. However, the use of SSRs is far from disappearing because of their popular application in genetic diversity monitoring (Flanagan & Jones, 2019; Harrison et al., 2014; Lemopoulos et al., 2019; Vieira et al., 2016). These applications are characterized by low requirements regarding marker numbers and the long‐term scanning of the population. In such conditions, multiplex SSR‐PCR is the best choice considering the advantages of its low cost, the high locus polymorphism, and the convenience of its use (Sint, Raso, & Traugott, 2012).

Typical multiplex SSR‐PCR is characterized by the high locus polymorphism, the conservation of flanking sequences, specificity of the primers in the genome, and compatibility of primers. Typical methods for the development of multiplex SSR‐PCR assays mainly consist of five steps: the selection of a reference, repeat motif detection, primer design, validation of locus polymorphism, and primer compatibility validation (Andrés & Bogdanowicz, 2011; Kijas, Fowler, Garbett, & Thomas, 1994; Norris, Bradley, & Cunningham, 2000). These steps are time‐ and cost‐intensive, especially the validation of locus polymorphism of and the compatibility of primer pairs (Neff, Fu, & Gross, 2000). In addition, variations occurring at primer‐binding sites may cause null alleles, and primers with low specificity can lead to allele errors. With the development of sequencing technology and the accumulation of abundant sequences, the development of SSRs has shifted from the use of limited genome sequences from a single individual to the use of genome sequences from multiple individuals at the genome scale. With advances in next‐generation and third‐generation sequencing technology, reference assemblies, resequencing reads, and transcriptome reads can now be easily obtained. Recently, the in silico mining of polymorphic SSRs was proposed. However, the available tools are all lacking in some regards. PolyMorphPredict (Das et al., 2019) and PSR (Cantarella & D'Agostino, 2015) lack the masking of mutations in primer design. PolySSR (Tang et al., 2008) and CandiSSR (Xia et al., 2015) depend on assembled sequences. iMSAT (Andersen & Mills, 2014) is dependent on (insertion and deletion) Indel calling and lacks the masking of mutations in primer design. All of these programs output polymorphic SSRs at most and lack the assessment of the saturation of the allele number range and the potential for the development of multiplex PCR.

Based on the availability of reference assemblies and resequencing data, we conducted a systemic assessment of the development of multiplex SSR‐PCR assays directly from resequencing data. This strategy takes full advantage of genome information from populations at the whole‐genome scale with limited costs. The main issues that need to be assessed are the influence of genotype error on the selection of highly polymorphic loci and the estimation of allele length range. We first assessed the distribution of tandem repeats in the reference genome, the influence of genotyping error on the selection of highly polymorphic loci, and the estimation of allele length range. Then, a pipeline referred to as MultiplexSSR was established. Furthermore, groups of multiplex SSR‐PCR assays were validated.

## 2 MATERIALS AND METHODS

### 2.1 Sample collection and resequencing

**Figure 1:**
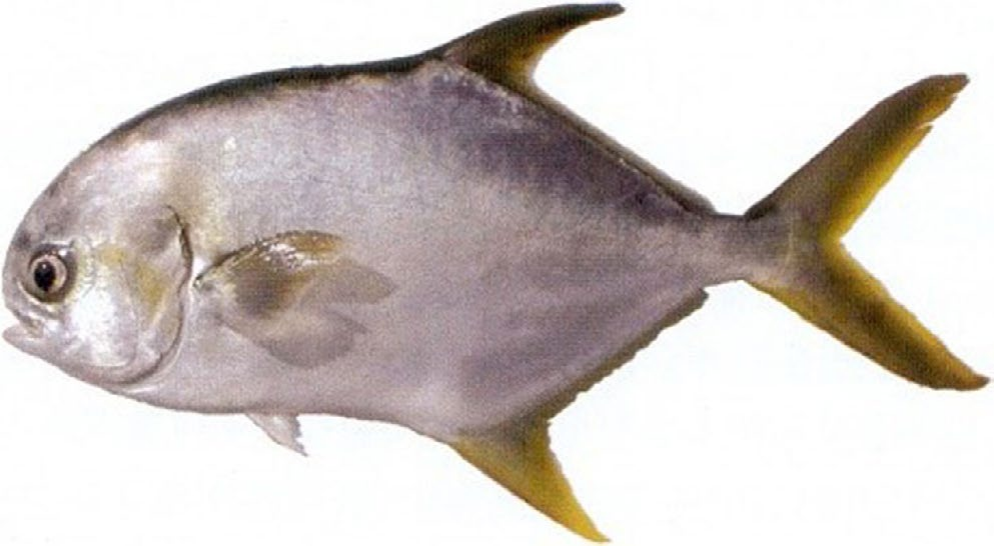
The species golden pompano (*Trachinotus ovatus*) used in developing this pipeline

The species golden pompano (*Trachinotus ovatus*) was used as a case study (Figure 1). A reference genome has been assembled for this species (GenBank Accession No.: GCA_900231065.1), which is a draft genome with a total length of 652 Mbp and a scaffold N50 of 1.67 Mbp. A full‐sib family (F201803) was sampled, including the parents and 100 offspring, to investigate the characteristics of the SSR genotype and allelotype error. Ten individuals (Pr) were sampled from the core collection (Guo et al., 2018) to develop multiplex SSR‐PCR assays. A mass cross‐population (PM2018) including 1819 offspring was collected to validate the multiplex SSR‐PCR primers. The full‐sib family (F201803) was included in this mass cross‐population.

A fin sample was cut from each individual and preserved in alcohol at −20°C. DNA extraction and quality testing were performed as previously described (Guo et al., 2018). The samples from the full‐sib family and ten randomly selected individuals were subjected to resequencing on the Illumina NovaSeq platform at the Novogene Bioinformatics Institute (Beijing, China).

### 2.2 Characteristics of tandem repeats

The characteristics of the tandem repeats in the reference genome were investigated. The repeat sequences in the golden pompano reference were detected using Tandem Repeats Finder version 4.07b (TRF) (Benson, 1999) with the recommended parameters. The range of the detected motif lengths was from 1 to 2,000, and the minimum repeat numbers for motif lengths of 2, 3, 4, 5, and 6 were 12, 8, 6, 5, and 4, respectively. Single sequence repeats were counted using the standard described by Jurka and Pethiyagoda (1995), under which single sequence repeats are categorized into 501 patterns, including two monomeric, four dimeric, 10 trimeric, 33 tetrameric, 102 pentameric, and 350 hexameric patterns. The patterns in the reference genome were counted with an Perl script.

### 2.3 Error profiles of the genotypes and allelotypes of single sequence repeats

In the full‐sib family (F201803), loci showing the same genotypes in the parents were selected to assess genotype and allelotype error. The genotypes of the offspring were expected to be the same as those of the parents.

First, the membership of the full‐sib family was confirmed with SNPs. The raw reads from the full‐sib family (F201803) were filtered using Trimmomatic v0.38 (Bolger, Lohse, & Usadel, 2014) with default parameters. The clean reads were mapped to the reference with BWA‐MEM v0.7.17 (Li, 2013). SAM and BAM format files were manipulated with SAMtools v1.9 (Li et al., 2009). The accurate mapped reads were selected for genotyping. GATK v4.1.1.0 (McKenna et al., 2010) was used to mark duplications with the MarkDuplicates tool and call SNPs with the Haplotypecaller tool. GATK VariantFiltration was used to implement hard filtering for SNPs (QUAL < 5,000, depth >3,000, quality by depth < 2, phred‐scaled Fisher's exact test *p* value > 60, root mean square mapping quality < 30, mapping quality Mann–Whitney rank‐sum < −10, mapping quality Mann–Whitney rank‐sum > 10, read position Mann–Whitney rank‐sum < −10, read position Mann–Whitney rank‐sum > 10, strand odds ratio > 3). Parenthood in the full‐sib family (F201803) was confirmed with Lep‐MAP3 (Rastas, 2017).

**Figure 2:**
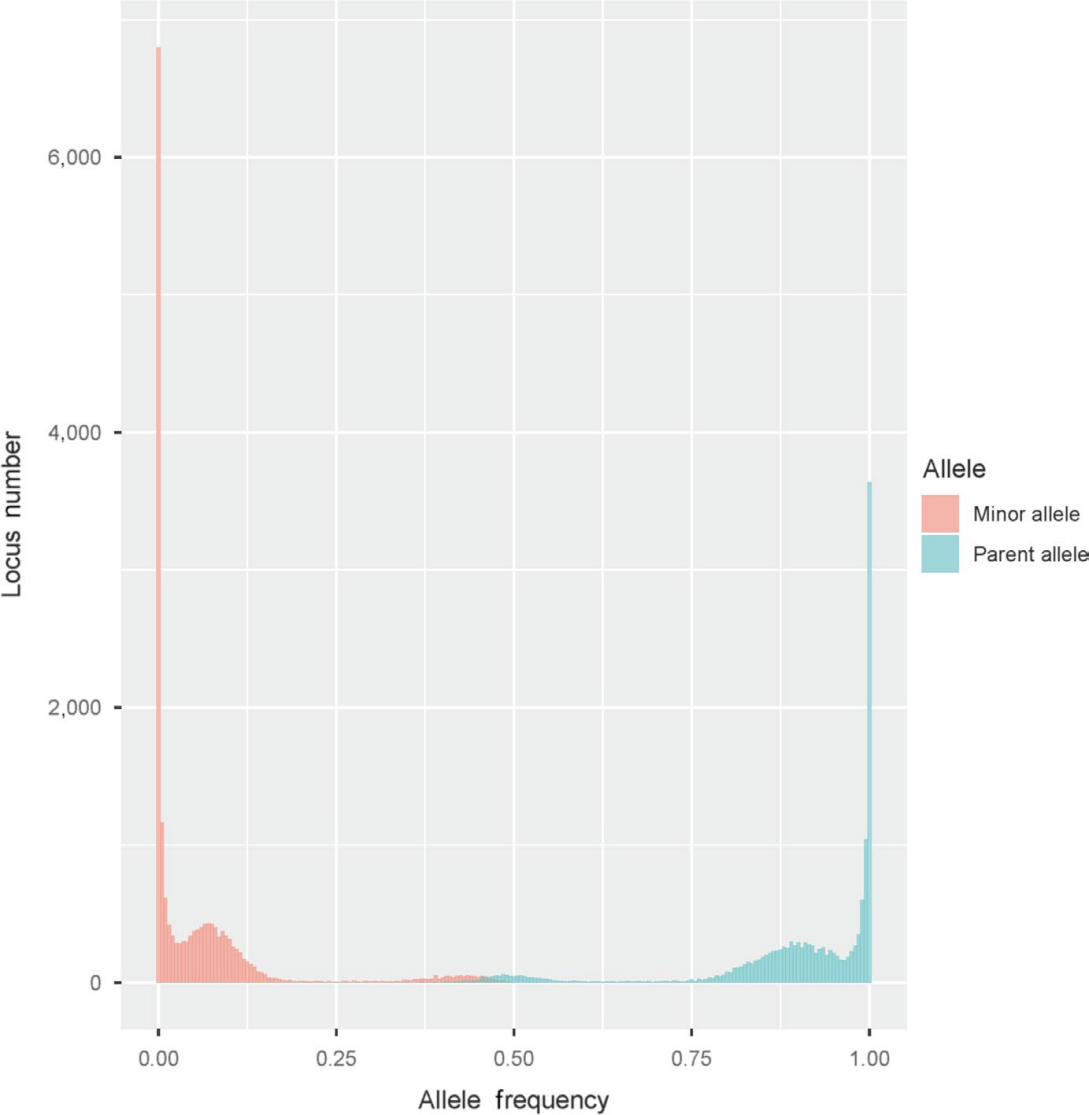
The histogram of SSR numbers before filtering in the full‐sib family (F201803). To estimate genotype error, the loci were filtered with four constraints: the depth in parents, the same genotypes in parents, the parental allele frequency in offspring, and the minor allele frequency in offspring. Error still existed under only the first two constraints. If the four alleles in the parents included one erroneous allele, the offspring were expected to exhibit the reference and minor alleles at frequencies of 0.75 and 0.25, respectively. If the four alleles in the parents included two error alleles, the parental allele and minor allele frequencies in the offspring were expected to be 0.50 and 050, respectively. Thus, loci with parental allele and minor allele frequency thresholds of 0.80 and 0.20 were selected as thresholds

Then, the loci confirmed to show the same genotypes in the parents were selected. The identified single sequence repeats with motif lengths of 2 to 6 were genotyped using lobSTR version 4.0.6 (Gymrek et al., 2012) with the options min‐het‐freq = 0.2, min‐border = 5, min‐bp‐before‐indel = seven, maximal‐end‐match = 15, and min‐read‐end‐match = 10. Only reads with a unique best map location and a read pair distance within 1,000 bp were considered in SSR calling; in this process, duplications were automatically excluded. The SSRs showing the same genotypes in the parents, a depth in each parent of greater than 10 (genotype error rate of approximately 10% and allelotype error rate of approximately 5%, Gymrek et al., 2012), a depth in all individuals of less than 900, a percentage of missing in the offspring of less than 40%, a parental allele frequency in the offspring of greater than 0.80, and a minor allele frequency in the offspring of less than 0.20 were selected (Figure 2). Unexpected genotypes in the offspring were treated as errors.

Third, the error profiles were investigated. A score was calculated for each genotype,

(1) $$S=\sum_{k=1}^{2}|A^{k}-R|/M$$

where *S*, score of the genotype; *A*, allele length; *R*, parental allele length; and *M*, motif length.

The Pearson correlation coefficient was calculated to estimate the relationships between motif length, the number of repeats, depth, and score using the psych package (Revelle, 2018). Furthermore, the genotype and allelotype error rates were calculated.

### 2.4 Estimation of allele numbers, maximum allele length, minimum allele length, and the allele length range

The ten randomly sampled individuals (Pr) (Guo et al., 2018) were also sequenced on the Illumina's NovaSeq platform as described above. SSRs were genotyped via the same approach used for the full‐sib family. The influence of the number of individuals on the estimation of locus polymorphism and the saturation of the allele length range was assessed. The loci that existed in all ten individuals and exhibited at least six alleles were selected. To simulate an increase in the number of individuals from 1 to 10, the genotypes at each locus were randomly drawn from one to ten individuals without replacement. The allele number, maximum allele length, minimum allele length, and allele length range for number of individuals were classified into ten groups. The differences between multiple joint groups were compared with the Friedman test, and pairwise comparisons between groups were assessed with the Nemenyi post hoc test (Nemenyi, 1963; Pohlert, 2014). This test was developed to account for family‐wise error and is a conservative test. Thus, the *p* values were not adjusted in pairwise comparisons.

### 2.5 Pipeline establishment and multiplex SSR‐PCR assay development

The pipeline was established based on the following principles. First, highly polymorphic SSRs at the whole‐genome scale should be selected. Second, the primers need to be specific and stable. Third, the designed multiplex SSR‐PCR primers can be labeled and used directly on the capillary platform.

**Figure 3:**
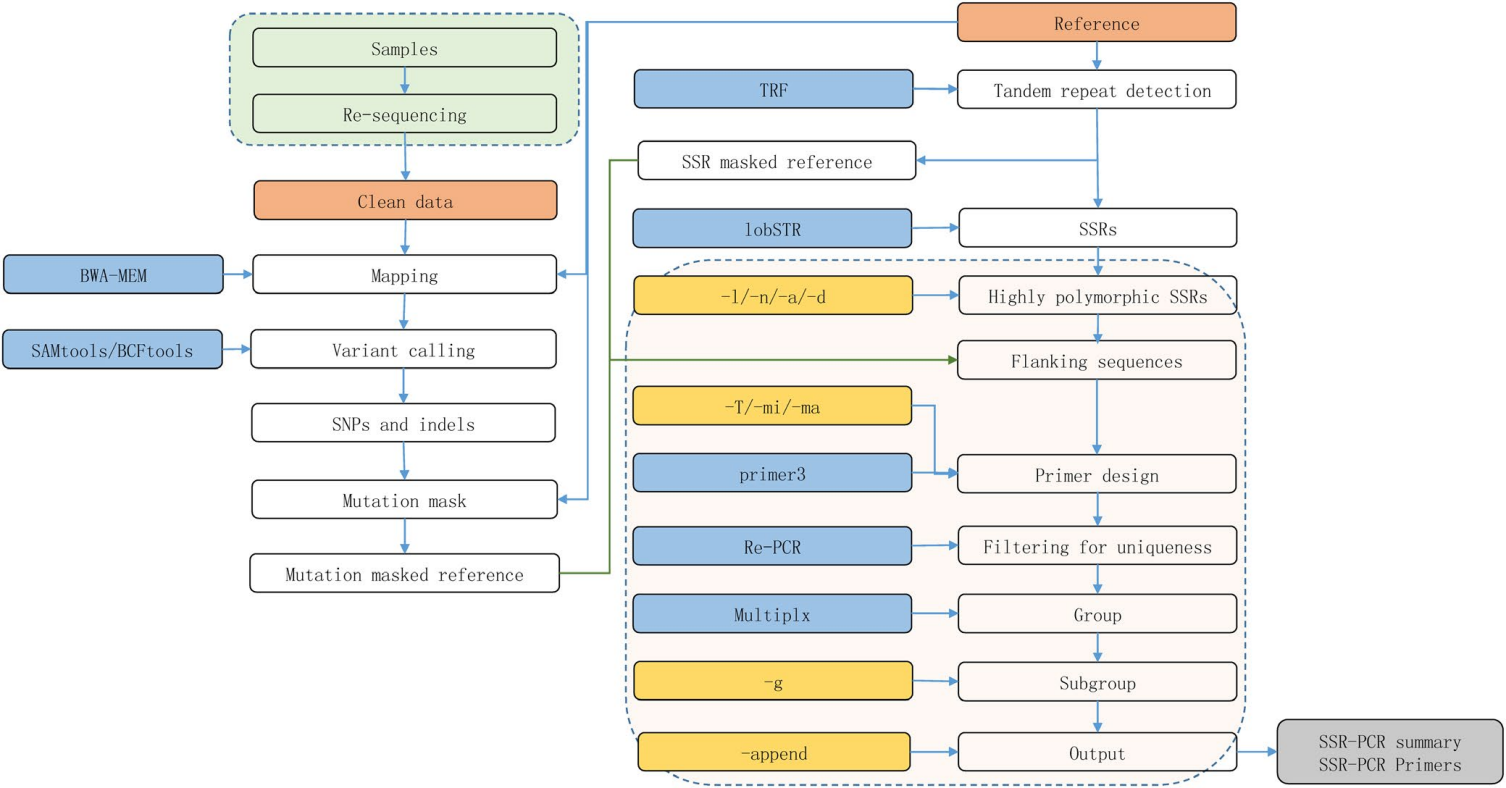
Proposed MultiplexSSR pipeline workflow. The pipeline takes resequencing data in fastq format and reference as the input and finally outputs multiplex PCR primers. The programs (blue background) TRF, BWA‐MEM, SAMtools, lobSTR, Primer3, Re‐PCR, and MultiPLX are integrated. The parameters (yellow background), the optimum annealing temperature (‐T), minimum length of repeat units (‐l), the minimum number of genotyped individuals (‐l), the minimum number of alleles (‐a), the minimum depth (‐d), the minimum length of the amplicon (‐mi), the maximum length of the amplicon (‐ma), and the minimum space between SSRs (‐g) can be adjusted. This pipeline can be used in beginning‐to‐end model or skip model by skipping read mapping and genotyping (‐s)

A pipeline referred to as MultiplexSSR (Figure 3) was established to develop multiplex SSR‐PCR assays from resequencing data. The raw reads were filtered with Trimmomatic v0.38 (Bolger et al., 2014) with format parameters. SSRs were called with lobSTR (Gymrek et al., 2012) as indicated above. The primers were designed with Primer3 (Untergasser et al., 2012) with an optimal primer length of 21 bp. To reduce the negative influence of mutation on the binding sites of the primers in the template, SNPs and Indels were called using BWA‐MEM (Li, 2013), SAMtools, and BCFtools (Li et al., 2009), and these mutations were replaced with “N” in the reference. The uniqueness of the binding sites of the primer pairs in the reference was evaluated using re‐PCR (Schuler, 1998) with the maximum allowed mismatches and the number of Indels per primer set to 3. Primer pairs were assessed for compatibility and grouped using MultiPLX (Kaplinski, & Remm, 2015) with “normal” stringency. The primer pairs in the same group were further divided into subgroups based on the allele length range and their position so that the primer pairs in the same subgroup could be labeled with the same fluorochrome.

The resequencing data from ten randomly selected individuals (Pr) were used to test the pipeline. The optimum annealing temperature, minimum length of a repeat unit, minimum number of genotyped individuals, minimum number of alleles, minimum depth of the genotype, minimum length of the amplicon, maximum length of the amplicon, and minimum space were set to 60°C, 3, 5, 5, 1, 80 bp, 480 bp, and 20 bp, respectively.

### 2.6 Validation and application

Six groups of primer pairs were selected for the validation of primers based on the locus number in each subgroup, and two of these groups were used for pedigree construction. To reduce cost, a universal primer‐multiplex PCR method was employed (de Arruda, Gonçalves, Schneider, Da, & Morielleversute, 2010; Ge, Cui, Jing, & Hong, 2014; Steffens, Sutter, & Roemer, 1993; Sudo et al., 2018). For convenience, only two subgroups from each group were selected. The ratio of labeled forward primer: reverse primer: dye‐labeled universal primer was 1:4: 4 (de Arruda et al., 2010; Ge et al., 2014; Schuelke, 2000; Steffens et al., 1993; Sudo et al., 2018). The primers were synthesized by Ruiboxingke Biotech. Co. Ltd. PCR amplification was performed using Premix Taq™ Hot Start Version (Takara, Cat. # R028A) with the following program: 3 min at 98°C; 35 cycles of 10 s at 98°C, 40 s at 57°C, and 60 s at 72°C; 15 cycles of 10 s at 98°C, 40 s at 53°C, and 60 s at 72°C; and 10 min at 72°C. The PCR products were tested via 1% agarose gel electrophoresis and genotyped in a 3730XL capillary DNA analyzer (Applied Biosystems) at Ruiboxingke Biotech. Co. Ltd. The allele size was analyzed using GeneMapper version 4.0 (Applied Biosystems) and verified manually. The summary statistics of the SSRs were obtained with GenAlEx version 6.5 (Peakall & Smouse, 2012). The pedigree was reconstructed using Colony 2.0.6.4 with the genotyping error set at 0.05 (Jones & Wang, 2010).

## 3 RESULTS

**Figure 4:**
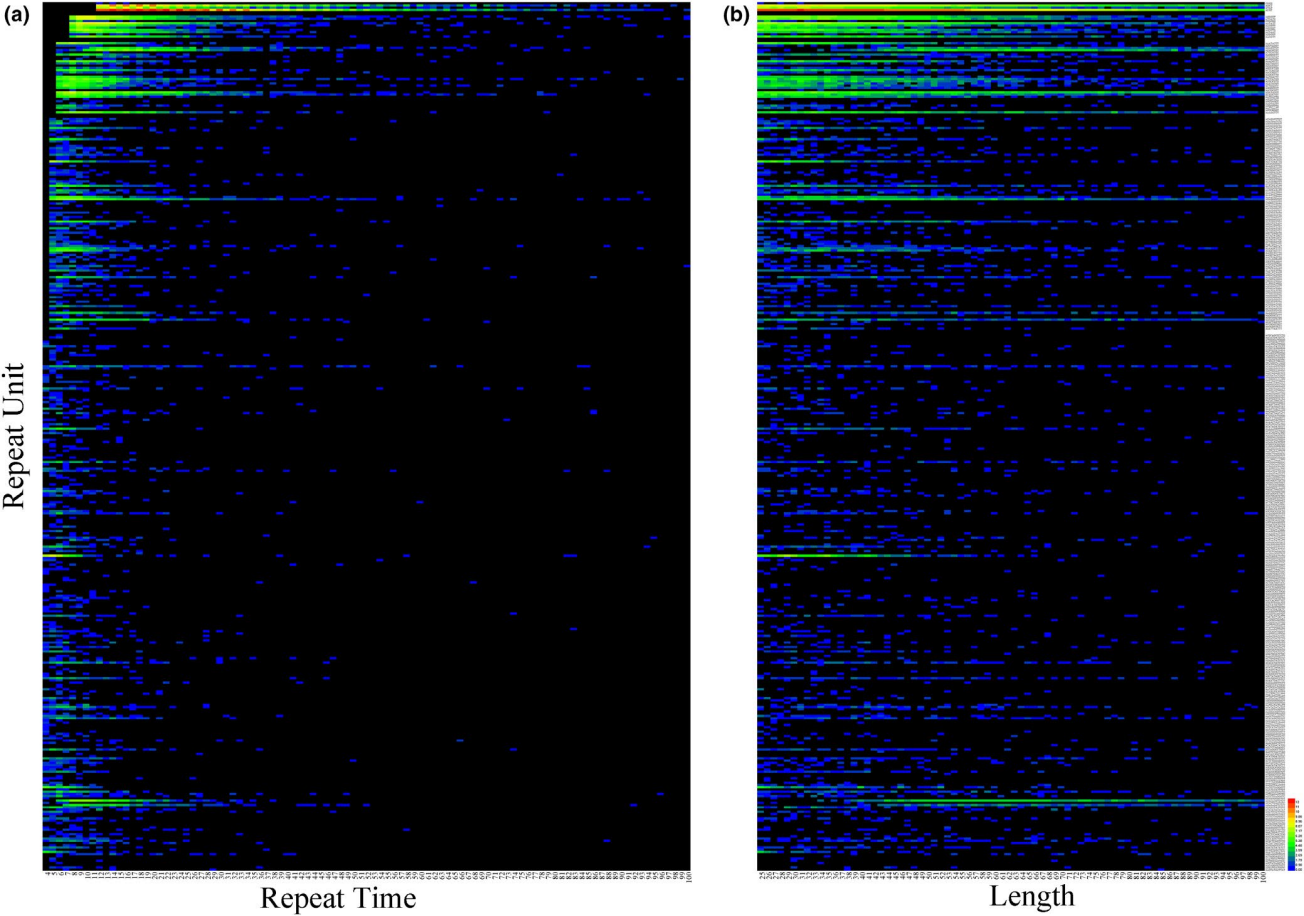
Heat map of single sequence repeats in the golden pompano assembly. The single sequence repeats are counted and catalogued by the number of repeats (a) or length (b)

Reads of 150 bp captured most of the SSRs. Tandem repeats in the genome sequence of golden pompano were searched. The total length of the tandem repeats was 15.7 Mbp, accounting for 2.5% of the reference genome sequence. The motif length of the tandem repeats ranged from 1 to 503. The total number of tandem repeats was 227,774, among which SSRs with motif lengths of 2–6 and 3–6 accounted for 56.1% and 13.4% of the repeats, respectively. Most of the SSRs contained fewer than 20 repeat units (66.0%, Figure 4a) and were less than 100 bp (98.0%, Figure 4b).

**Figure 5:**
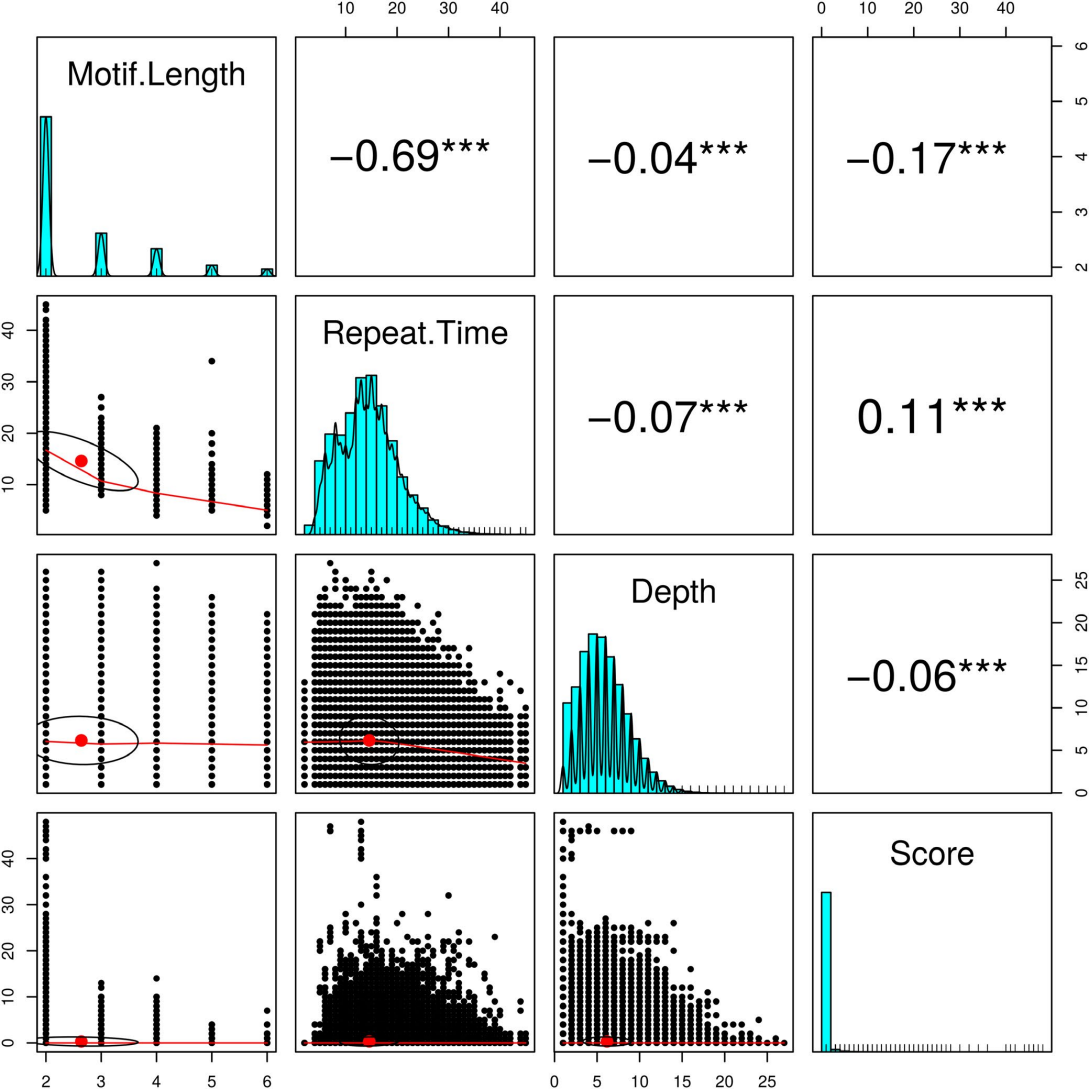
The scatter plots of the matrices of motif length, the number of repeats, depth, and score are shown, with bivariate scatter plots below the diagonal, histograms on the diagonal, and the Pearson correlation above the diagonal. The highest Pearson correlation between score and motif length suggests that motif length has the largest impact on genotyping accuracy. Repeat time and depth are also negatively and positively influenced by genotype accuracy, respectively

The error of allelotyping was less than that of genotyping. The average depth for the parents in the full‐sib family (F201803) was 33.37‐fold (Table 1). After filtering, 754,511 SNPs and 16,348 SSRs were obtained, and the full‐sib family was confirmed. The parameter score (Formula 1) was used to measure genotype error. The score was significantly correlated with motif length, the number of repeats, and depth, with the Pearson correlation coefficients of −0.17, 0.1,1 and −0.06, respectively (Figure 5). The total error rates of the genotypes and allelotypes were 17.37% and 10.90%, respectively, among which dimeric patterns accounted for 92.88% and 94.98%, respectively (Table 2). Because of the high error rate, the dimeric patterns were excluded in the following steps. Hence, the genotype error and allelotype error were reduced to 2.28% and 1.54%, respectively (Table 3). The alleles showing a length difference of greater than 10 bp because of error only accounted for 0.07% of all alleles.

**Table 1:** The data from the Illumina platform for each sample

	F201803		Random individuals (Pr)
	Parents	Offspring	
Average read pairs (×10^6^)	88.30	31.09 ± 4.95	49.14 ± 5.26
Average depth (whole genome)	33.37	13.05 ± 2.05	21.36 ± 2.32
Average depth (SNP)	29.53 ± 10.33	21.61 ± 12.79	/
Average depth (SSR)	17.46 ± 4.42	6.16 ± 2.84	4.54 ± 3.03

**Table 2:** The error rate for all SSRs (F201803)

Motif length	2	3	4	5	6	Sum
Genotype number	1,046,109	281,872	179,907	71,481	45,936	1,625,305
Allele number	2,092,218	563,744	359,814	142,962	91,872	3,250,610
Genotype error number	269,088	4,792	7,018	731	670	282,299
Genotype error rate in each catalogue	25.72%	1.70%	3.90%	1.02%	1.46%	17.37%
Genotype error rate in all genotypes	16.56%	0.29%	0.43%	0.04%	0.04%	17.37%
Genotype error rate in all error genotypes	95.32%	1.70%	2.49%	0.26%	0.24%	100.00%
Allelotype error number	336,620	6,295	9,600	989	905	354,409
Allelotype error rate in each catalogue	16.09%	1.12%	2.67%	0.69%	0.99%	10.90%
Allelotype error rate in all alleles	10.36%	0.19%	0.30%	0.03%	0.03%	10.90%
Allelotype error rate in all error genotypes	94.98%	1.78%	2.71%	0.28%	0.26%	100.00%
Allelotype error with a length difference >10 bp in all alleles	6,710	375	425	4	48	7,562
Allelotype error rate with a length difference >10 bp in all alleles	0.21%	0.01%	0.01%	0.00%	0.00%	0.23%
Allelotype error with an extra length difference >20 bp	794	3	8	0	1	806
Allelotype error rate with a length difference >20 bp in all alleles	0.02%	0.00%	0.00%	0.00%	0.00%	0.02%

**Table 3:** The error rate without dimeric patterns (F201803)

Motif length	3	4	5	6	Sum
Genotype number	281,872	179,907	71,481	45,936	579,196
Allele number	4,792	7,018	731	670	13,211
Genotype error number	1.70%	3.90%	1.02%	1.46%	2.28%
Genotype error rate in each catalogue	0.83%	1.21%	0.13%	0.12%	2.28%
Genotype error rate in all genotypes	36.27%	53.12%	5.53%	5.07%	100.00%
Genotype error rate in all error genotypes	6,295	9,600	989	905	17,789
Allelotype error number	375	425	4	48	852
Allelotype error rate in each catalogue	5.96%	4.43%	0.40%	5.30%	4.79%
Allelotype error rate in all the alleles	0.54%	0.83%	0.09%	0.08%	1.54%
Allelotype error rate in all error alleles	44.01%	49.88%	0.47%	5.63%	100.00%
Allelotype error with a length difference >10 bp in all alleles	0.03%	0.04%	0.00%	0.00%	0.07%
Allelotype error rate with a length difference >10 bp in all alleles	3	8	0	1	12
Allelotype error with a length difference >20 bp in all alleles	0.00%	0.00%	0.00%	0.00%	0.00%
Allelotype error rate with a length difference >20 bp in all alleles	563,744	359,814	142,962	91,872	1,158,392

**Figure 6:**
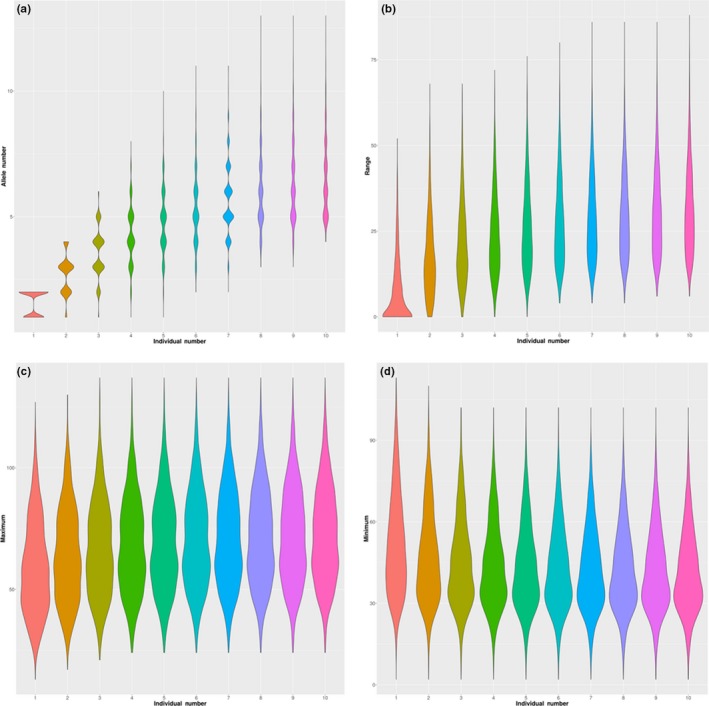
The increasing trends of allele number (a), allele length range (b), maximum allele length (c), and minimum allele length (d) depending on the number of individuals

The allele length range was nearly saturated for 10 individuals. The influence of the number of individuals on the allele number, allele length range, maximum allele length, and minimum allele length was investigated (Figure 6 and Tables 4 and 5). The number of individuals significantly affected all four parameters (*p* value < 2.2 × 10^–16^). The allele length range reached saturation, while the allele number was still increasing. The maximum allele length, minimum allele length, and allele length range were not significantly different in the groups with 9 and 10 individuals (*p* value > .05), while the allele number was significantly different between all the groups with different numbers of individuals (*p* value < .05).

**Table 4:** *p* Values of the Nemenyi post hoc test between groups with different numbers of individuals for the allele number (below) and allele length range (above) in the randomly selected individuals (Pr)

	1	2	3	4	5	6	7	8	9	10
1		<2 × 10^–16^	<2 × 10^–16^	<2 × 10^–16^	<2 × 10^–16^	<2 × 10^–16^	<2 × 10^–16^	<2 × 10^–16^	<2 × 10^–16^	<2 × 10^–16^
2	8.6 × 10^–14^		2.7 × 10^–14^	<2 × 10^–16^	<2 × 10^–16^	<2 × 10^–16^	<2 × 10^–16^	<2 × 10^–16^	<2 × 10^–16^	<2 × 10^–16^
3	<2 × 10^–16^	9.8 × 10^–14^		1.2 × 10^–13^	<2 × 10^–16^	<2 × 10^–16^	<2 × 10^–16^	<2 × 10^–16^	<2 × 10^–16^	<2 × 10^–16^
4	<2 × 10^–16^	<2 × 10^–16^	1.1 × 10^–13^		1.1 × 10^–10^	<2 × 10^–16^	<2 × 10^–16^	<2 × 10^–16^	<2 × 10^–16^	<2 × 10^–16^
5	<2 × 10^–16^	<2 × 10^–16^	<2 × 10^–16^	9.8 × 10^–14^		3.7 × 10^–06^	8.6 × 10^–14^	<2 × 10^–16^	<2 × 10^–16^	<2 × 10^–16^
6	<2 × 10^–16^	<2 × 10^–16^	<2 × 10^–16^	<2 × 10^–16^	1.1 × 10^–13^		.0016	1.6 × 10^–13^	<2 × 10^–16^	<2 × 10^–16^
7	<2 × 10^–16^	<2 × 10^–16^	<2 × 10^–16^	<2 × 10^–16^	<2 × 10^–16^	3.6 × 10^–12^		.0029	1.3 × 10^–12^	9.9 × 10^–14^
8	<2 × 10^–16^	<2 × 10^–16^	<2 × 10^–16^	<2 × 10^–16^	<2 × 10^–16^	<2 × 10^–16^	1.4 × 10^–09^		.0103	9.7 × 10^–09^
9	<2 × 10^–16^	<2 × 10^–16^	<2 × 10^–16^	<2 × 10^–16^	<2 × 10^–16^	<2 × 10^–16^	<2 × 10^–16^	2.5 × 10^–07^		**.1684**
10	<2 × 10^–16^	<2 × 10^–16^	<2 × 10^–16^	<2 × 10^–16^	<2 × 10^–16^	<2 × 10^–16^	<2 × 10^–16^	9.5 × 10–14	.00027	

Note

*p* values larger than .05 are shown in bold.

**Table 5:** *p* values of the Nemenyi post hoc test between groups with different numbers of individuals for the maximum allele length (below) and minimum allele length (above) in the randomly selected individuals (Pr)

	1	2	3	4	5	6	7	8	9	10
1		<2 × 10^–16^	<2 × 10^–16^	<2 × 10^–16^	<2 × 10^–16^	<2 × 10^–16^	<2 × 10^–16^	<2 × 10^–16^	<2 × 10^–16^	<2 × 10^–16^
2	9.4 × 10^–14^		1.9 × 10^–11^	<2 × 10^–16^	<2 × 10^–16^	<2 × 10^–16^	<2 × 10^–16^	<2 × 10^–16^	<2 × 10^–16^	<2 × 10^–16^
3	<2 × 10^–16^	7.9 × 10^–14^		2.7 × 10^–05^	8.9 × 10^–14^	1.8 × 10^–14^	<2 × 10^–16^	<2 × 10^–16^	<2 × 10^–16^	<2 × 10^–16^
4	<2 × 10^–16^	<2 × 10^–16^	1.2 × 10^–07^		.01125	1.7 × 10^–08^	1.3 × 10^–13^	8.8 × 10^–14^	<2 × 10^–16^	<2 × 10^–16^
5	<2 × 10^–16^	<2 × 10^–16^	2.0 × 10^–14^	5.9 × 10^–06^		**.19682**	.00023	2.0 × 10^–09^	8.6 × 10^–14^	8.3 × 10^–14^
6	<2 × 10^–16^	<2 × 10^–16^	<2 × 10^–16^	1.1 × 10^–13^	.0035		**.66735**	.00321	9.4 × 10^–08^	1.1 × 10^–11^
7	<2 × 10^–16^	<2 × 10^–16^	<2 × 10^–16^	<2 × 10^–16^	5.7 × 10^–11^	.0479		**.56966**	.00178	2.7 × 10^–06^
8	<2 × 10^–16^	<2 × 10^–16^	<2 × 10^–16^	<2 × 10^–16^	9.5 × 10^–14^	1.2 × 10^–07^	**.1472**		**.56544**	.02563
9	<2 × 10^–16^	<2 × 10^–16^	<2 × 10^–16^	<2 × 10^–16^	<2 × 10^–16^	9.3 × 10^–14^	4.3 × 10^–06^	**.2342**		**.94734**
10	<2 × 10^–16^	<2 × 10^–16^	<2 × 10^–16^	<2 × 10^–16^	<2 × 10^–16^	8.9 × 10^–14^	2.2 × 10^–12^	8.1 × 10^–05^	**.4545**	

Note

*p* values larger than .05 are shown in bold.

The designed primers exhibited a high rate of actual amplification. When MultiplexSSR was applied to the ten randomly selected individuals (Pr), 49 groups of primers were designed (Tables S1 and S2). The number of primer pairs in each group ranged from 9 to 11. Six groups of primers (G10, G20, G25, G36, G42, and V1) were selected to validate their efficacy in actual amplification. These groups contained 55 loci, 47 of which could be stably amplified (Figure S1, Tables S3 and S4 and Data S1).

The application of the designed multiplex SSR‐PCR assays validated the robustness of MultiplexSSR. Two groups of primers (Table S5, G36 and V1) corresponding to 13 SSRs were selected for pedigree construction in the mass cross‐population (PM2018). The overall expected heterozygosity and observed heterozygosity were 0.607 and 0.738, respectively (Table S5). After filtering out individuals with fewer than seven loci and families with fewer than 10 offspring, the remaining 1726 offspring were assigned to six full‐sib families (Figure S2). The assignment results for the members in the full‐sib family (F201803) were completely consistent with different methods.

## 4 DISCUSSION

In this study, the distribution of single sequence repeats and errors of SSR genotyping and allelotyping and the influence of the number of individuals on the maximum allele length, minimum allele length, and allele length range were assessed. Based on these results, a pipeline referred to as MultiplexSSR was developed, which fills the gap between next‐generation sequencing technology and multiplex SSR‐PCR assays. The selected loci were highly polymorphic at the whole‐genome scale, and the primers exhibited a high validation rate and were grouped according to compatibility, allele length range, and allele length.

### 4.1 Repeat pattern selection

Recently, the most popular next‐generation sequencing platform has been that of Illumina, which produces 150‐bp paired‐end reads and performs PCR amplification for library construction. These two characteristics determine the feasibility and difficulty of SSR genotyping. The read length is sufficiently long to capture most single sequence repeats, even though the amplification step introduces a large amount of error. Among single sequence repeats, 98.0% are less than 100 bp in length, which is shorter than the single‐end length of the read pairs considering that the reads must span the whole repeat region (Gymrek et al., 2012; Highnam et al., 2013) and the read quality filtering procedure. The genotype and allelotype errors are 17.37% and 10.90%, respectively, among which dimeric patterns account for 92.88% and 87.40%, respectively. In addition, the genotyping of SSRs on a capillary platform or a next‐generation sequencing platform requires PCR amplification (Barbian et al., 2018; De Barba et al., 2017; Li et al., 2017; Šarhanová, Pfanzelt, Brandt, Himmelbach, & Blattner, 2018; Vartia et al., 2016; Zhan et al., 2017), which leads to the same dilemma of a higher error rate in dimeric patterns (Weber & Broman, 2000; Yue & Xia, 2014). When there is a large quantity of candidate SSRs, dimeric patterns can be filtered out, even at the expense of losing higher polymorphism in these patterns (Gymrek et al., 2012; Yue & Xia, 2014). Alternatively, the use of the PCR‐free model on the Illumina platform or PCR‐Free DNBseq technology can reduce the error caused by PCR amplification at the sacrifice of cost.

### 4.2 Requirements of depth and the number of individuals

The depth is a critical parameter for SSR and SNP genotyping. The genotype and allelotype errors decrease with increasing depth (Guo et al., 2017; Gymrek et al., 2012). The parameter scores obtained in our study were significantly and negatively correlated with depth (Figure 5). Even though high depth can improve the accuracy of genotypes and allelotypes, our results demonstrated that a low depth can still meet the requirements for the selection of highly polymorphic loci and the estimation of allele length range. First, we adopted the allele number instead of heterozygosity to screen out highly polymorphic loci. The allele number, allele richness, and expected heterozygosity (Allendorf, 1986; Chistiakov, Hellemans, & Volckaert, 2006; Guo et al., 2018) are generally used to assess locus polymorphism. Here, the allele number was used considering that allelotypes are characterized by a lower error rate than genotypes (Tables 2 and 3) and that the allele numbers from certain individuals are more sensitive to diversity (Loughnan et al., 2013). Second, the allele length range is only determined by the longest allele and shortest allele and is more closely related to the number of individuals than the depth in each individual. In our randomly selected individuals (Pr), the range tended to be fixed in groups of nine individuals or more, while the allele number was still increasing (Figure 6). Third, resequencing data are usually used to call SNPs, and these data always show genome coverage of less than 20‐fold. The use of low‐depth data could broaden the application of our pipeline. Fourth, the calling of SSRs requires a single‐end read spanning the repeat region, which reduces the actual depth. In the F201803 family, the depth of SSR genotypes was almost half of the depth of SNP genotypes and the whole genome (Table 1).

To design multiplex SSR‐PCR assays, the allele length range and allele position need to be accurately determined. The number of individuals is the most important parameter along with depth. In this study, the depth for the randomly selected samples (Pr) was approximately 20‐fold (Table 1). When the depth was preconditioned, the maximum allele length, minimum allele length, and allele range for each SSR tended to be fixed when the number of individuals was increased to 10 (Tables 4 and 5, Figure 6). However, the genetic diversity of golden pompano is relatively low (Guo et al., 2018), and the allele length range only approximated saturation when the group size reached ten individuals. For other species with high diversity, the inclusion of a greater number of individuals would be more appropriate. In the pipeline, the “random.pl” script provides the function of assessing the saturation of these statistics.

### 4.3 Validation rates of primers

In our pipeline, two approaches are applied to increase the validation rate. First, we take full advantage of resequencing data to mask the positions that contain mutations in the population. A primer binding to a DNA sequence is always blocked by a mutation located within the primer‐binding region (Tang et al., 2008). In the MultiplexSSR pipeline, the called raw SNPs and Indels were slightly filtered according only to a Phred quality of 20, and their masking screened out variant sites to the full extent via primer design. Second, in silico PCR (Schuler, 1998) was used to filter out primers with multiple targets. The maximum numbers of allowed mismatches and Indels per primer were both set to 3, which greatly increases the specificity of primers. In addition, the application of a consistent annealing temperature in primer design and the use of a hot‐start enzyme also improve the validation rate.

Our pipeline reduces the cost and labor required for genotyping, and the developed SSRs are more polymorphic. The designed primers could be amplified in at least 85% of cases, which is a relatively high percentage and guarantees the success of multiplex PCR. Previously, polymorphic SSRs were developed for an entire cultured golden pompano population via the traditional method. The expected heterozygosity and observed heterozygosity were 0.591 and 0.592, respectively (Guo et al., 2018), which are similar to the values obtained in an independent report (Lei & You‐Jun, 2011). In this study, the expected heterozygosity and observed heterozygosity for the mass cross‐population were 0.607 and 0.738, respectively, even with a extremely limited number of parents. In the previous studies, SSRs were genotyped individually (Guo et al., 2018; Lei & You‐Jun, 2011), whereas they were genotyped only in 2 groups of multiplex PCR assays in our mass cross‐population (PM2018).

### 4.4 Flaws of this pipeline

Even though this pipeline takes full advantage of whole‐genome resequencing, improves the efficiency of SSR‐PCR assay development, and avoids the common issues of traditional methods, some flaws still exist. First, this pipeline relies heavily on the efficiency of SSR genotyping, which leads to the filtering out of dimeric patterns. However, dimeric patterns are the most polymorphic and abundant type of SSRs (Table 2; Gymrek et al., 2012). Considering the acceptable cost of PCR‐free library construction for approximately a dozen individuals, the use of PCR‐free libraries can be attempted under the newly developed multiplex SSR‐PCR approach. Second, the presence of Indels in addition to the target SSRs within an amplicon will lead to alleles with noninteger repeats of the length of motifs, which are only masked to improve the quality of primers in this pipeline. As the occurrence rate of Indels, including SSRs (McMahon et al., 2017), is only approximately 0.1%, the actual occurrence of Indels in addition to SSRs will be rare. In such conditions, the abnormal allele needs to be explained with caution. Third, this pipeline depends heavily on published algorithms, especially LobSTR (Gymrek et al., 2012). Even though this algorithm shows excellent performance in SSR genotyping, the performance could be improved with popSTR (Kristmundsdóttir, Sigurpálsdóttir, Kehr, & Halldórsson, 2016) and STRScan (Tang & Nzabarushimana, 2017).

## CONFLICT OF INTEREST

None declared.

## AUTHOR CONTRIBUTIONS

L. Guo analyzed the data, developed the pipeline, and wrote the draft manuscript. Q. Yang and J. Yang validated the primers. N. Zhang collected ten randomly selected individuals, and L. Guo, N. Zhang, B. Liu, K. Zhu, and H. Guo collected the samples from the mass cross‐population. L. Guo, D. Zhang, and S. Jiang designed the pipeline. All the authors contributed to the manuscript revision.

## Data Availability

MultiplexSSR is a free open‐source program distributed under a GPLv3 license and is available at https://github.com/zsdxgl/MultiplexSSR. The “stastic.misa.motif.pl” and “stastic.misa.length100.pl” scripts are provided to count the SSR patterns. The “random.pl” script is also provided to test the saturation of the allele length range depending on an increasing number of individuals. The sequencing data for ten randomly collected samples (Pr) and the full‐sib family (F201803) were submitted to SRA under BioProject Accession Nos. PRJNA484082 and PRJNA552381, respectively.
