# Supplementary figures and images for: MultiplexSSR: A pipeline for developing multiplex SSR‐PCR assays from resequencing data
Source: Ecol Evol. 2020 Mar 4;10(6):3055–67. doi: 10.1002/ece3.6121 (PMC7083706; doi:10.1002/ece3.6121)

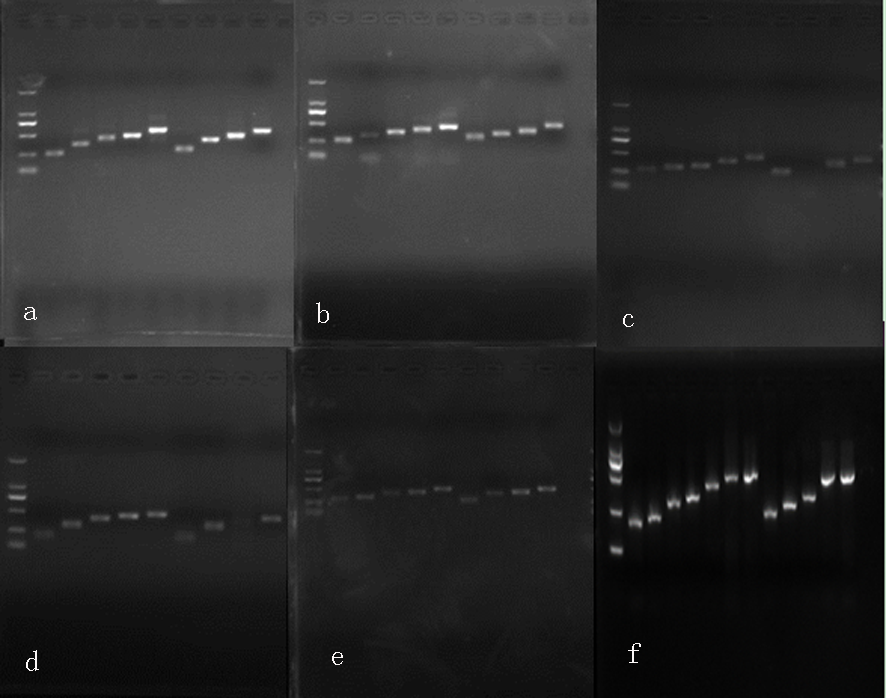

Supplement: Supplementary file 1 [file ECE3-10-3055-s001.tif]

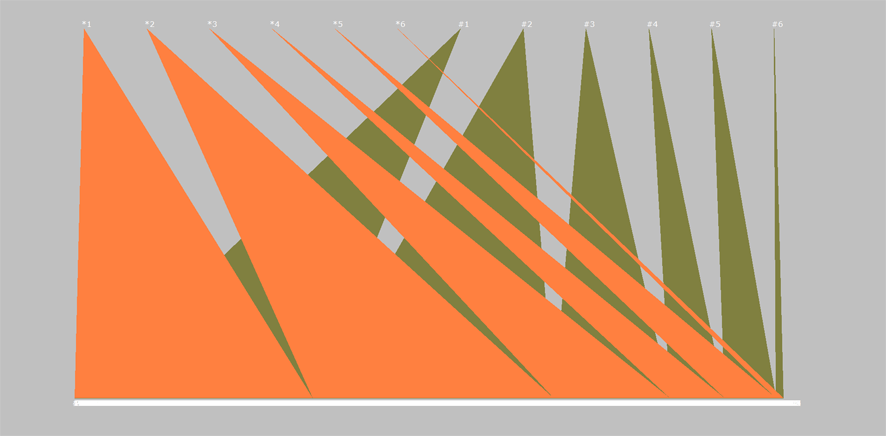

Supplement: Supplementary file 2 [file ECE3-10-3055-s002.tif]
