# Supplementary material for: MultiplexSSR: A pipeline for developing multiplex SSR‐PCR assays from resequencing data
Source: Ecol Evol. 2020 Mar 4;10(6):3055–67. doi: 10.1002/ece3.6121 (PMC7083706; doi:10.1002/ece3.6121)
Supplement: Supplementary file 5 [file ECE3-10-3055-s005.doc]

1. Multiplex SSR-PCR V1

| Item | | | Volume (ul) |
| --- | --- | --- | --- |
| BSA (2mg/ml) | | | 0.45 |
| TaKaRa Ex Taq HS | | | 7.5 |
| M13-FAM (10umol/L) | | | 0.84 |
| PQE-HEX (10umol/L) | | | 0.6 |
| FU | 10umol/L | Tov25439, Tov26724, Tov6129, Tov33340, Tov32776, Tov16774, Tov15555 | 0.06/each |
|  | 20umol/L | Tov19037, Tov34412, Tov4695, Tov31837 |  |
|  | 5umol/L | Tov32142 |  |
| R | 10umol/L | Tov25439, Tov26724, Tov6129, Tov33340, Tov32776, Tov16774, Tov15555 | 0.24/each |
|  | 20umol/L | Tov19037, Tov34412, Tov4695, Tov31837 |  |
|  | 5umol/L | Tov32142 |  |
| Template | | | 1.0 |
| Total | | | 15.0 |

1. Multiplex SSR-PCR G6

| Item | | | Volume (ul) |
| --- | --- | --- | --- |
| BSA (2mg/ml) | | | 0.45 |
| TaKaRa Ex Taq HS | | | 7.5 |
| RV3-ROX (10umol/L) | | | 1.2 |
| PVP-TAMAR (10umol/L) | | | 0.96 |
| FU | 10umol/L | Tov256, Tov356, Tov87, Tov416 | 0.06/each |
|  | 20umol/L | Tov74, Tov110, Tov340 |  |
|  | 100umol/L | Tov559, Tov240 |  |
| R | 10umol/L | Tov256, Tov356, Tov87, Tov416 | 0.24/each |
|  | 20umol/L | Tov74, Tov110, Tov340 |  |
|  | 100umol/L | Tov559, Tov240 |  |
| Template | | | 1.0 |
| Total | | | 15.0 |

1. Multiplex SSR-PCR G10

| Item | | | Volume (ul) |
| --- | --- | --- | --- |
| BSA (2mg/ml) | | | 0.45 |
| TaKaRa Ex Taq HS | | | 7.5 |
| RV3-ROX (10umol/L) | | | 1.2 |
| PVP-TAMAR (10umol/L) | | | 0.96 |
| FU | 10umol/L | Tov223, Tov210, Tov465, Tov379 | 0.06/each |
|  | 20umol/L | Tov428, Tov118, Tov389, Tov278 |  |
|  | 50umol/L | Tov32 |  |
| R | 10umol/L | Tov223, Tov210, Tov465, Tov379 | 0.24/each |
|  | 20umol/L | Tov428, Tov118, Tov389, Tov278 |  |
|  | 50umol/L | Tov32 |  |
| Template | | | 1.0 |
| Total | | | 15.0 |

1. Multiplex SSR-PCR G20.

| Item | | | Volume (ul) |
| --- | --- | --- | --- |
| G20 | | | 15.0 |
| BSA (2mg/ml) | | | 0.45 |
| TaKaRa Ex Taq HS | | | 7.5 |
| RV3-ROX (10umol/L) | | | 1.2 |
| PVP-TAMAR (10umol/L) | | | 0.96 |
| FU | 10umol/L | Tov2, Tov214, Tov464, Tov218, Tov553, Tov60 | 0.06/each |
|  | 20umol/L | Tov314, Tov448 |  |
|  | 50umol/L | Tov141 |  |
| R | 10umol/L | Tov2, Tov214, Tov464, Tov218, Tov553, Tov60 | 0.24/each |
|  | 20umol/L | Tov314, Tov448 |  |
|  | 50umol/L | Tov141 |  |
| Template | | | 1.0 |
| Total | | | 15.0 |

1. Multiplex SSR-PCR G36.

| Item | | | Volume (ul) |
| --- | --- | --- | --- |
| BSA (2mg/ml) | | | 0.45 |
| TaKaRa Ex Taq HS | | | 7.5 |
| M13-FAM (10umol/L) | | | 0.6 |
| PQE-HEX (10umol/L) | | | 0.48 |
| FU | 10umol/L | Tov368, Tov178, Tov82, Tov533 | 0.06/each |
|  | 20umol/L | Tov530, Tov425, Tov207, Tov91 |  |
|  | 6.7umol/L | Tov215 |  |
| R | 10umol/L | Tov368, Tov178, Tov82, Tov533 | 0.24/each |
|  | 20umol/L | Tov530, Tov425, Tov207, Tov91 |  |
|  | 6.7umol/L | Tov215 |  |
| Template | | | 1.0 |
| Total | | | 15.0 |

1. Multiplex SSR-PCR G42.

| Item | | | Volume (ul) |
| --- | --- | --- | --- |
| BSA (2mg/ml) | | | 0.45 |
| TaKaRa Ex Taq HS | | | 7.5 |
| M13-FAM (10umol/L) | | | 1.2 |
| PQE-HEX (10umol/L) | | | 0.96 |
| FU | 10umol/L | Tov25, Tov307, Tov92 | 0.06/each |
|  | 20umol/L | Tov18, Tov80, Tov400, Tov487 |  |
|  | 6.7umol/L | Tov357, Tov105 |  |
| R | 10umol/L | Tov25, Tov307, Tov92 | 0.24/each |
|  | 20umol/L | Tov18, Tov80, Tov400, Tov487 |  |
|  | 6.7umol/L | Tov357, Tov105 |  |
| Template | | | 1.0 |
| Total | | | 15.0 |
