# Supplementary material for: MultiplexSSR: A pipeline for developing multiplex SSR‐PCR assays from resequencing data
Source: Ecol Evol. 2020 Mar 4;10(6):3055–67. doi: 10.1002/ece3.6121 (PMC7083706; doi:10.1002/ece3.6121)
Supplement: Supplementary file 6 [file ECE3-10-3055-s006.doc]

SupTab 4. The primers used for validation.

| Group | Primer | Motif | Alleles | UF/R (from 5’ to 3’) |
| --- | --- | --- | --- | --- |
| G10 | Tov223 | GAG | 13 14 15 17 18 20 23 24 12 | AGCAAAATAGGCTGTCCCGCAGAGATCCCGAATTAAACAC/ TGCAGCTAAATTCCAGATCAGA |
|  | Tov32 | ATT | 20 21 22 23 27 28 16 | AGCAAAATAGGCTGTCCCCGCATGATAGCTTCTCCTTCTT/ GGAAATACTCACCACCCAGAGA |
|  | Tov210 | GTA | 19 20 21 22 23 24 25 28 14 | AGCAAAATAGGCTGTCCCAGGCTCCATACCACATAACCAT/ TTATCTCCAGGCACTTTCCAAT |
|  | Tov428 | TATC | 10 11 12 14 15 9 | AGCAAAATAGGCTGTCCCTTTATTCTCCATCAACCTGGCT/ TTCCATGTCAACAGCCACTTAC |
|  | Tov118 | AGAT | 9 11 12 13 14 16 17 19 7 | AGCAAAATAGGCTGTCCCCAGAGCTGTGCACCTTTAACAC/ TCCTTTGAAGTCTGCAGTGAAA |
|  | Tov465 | TTTC | 13 14 15 16 17 18 10 | GCCGACTTCGAGTTTGAGCCTTGCTCTATCTGATCCTGCT/ GTGAAGTTGTTGGCTACTGTGC |
|  | Tov389 | AGAT | 9 10 11 13 14 16 17 15 | GCCGACTTCGAGTTTGAGTTCTTGTTCATCACACAGTCCC/ CTCCACTCCAAATACTAACCGC |
|  | Tov379 | TTTCT | 5.6 9 10 11 13 7 | GCCGACTTCGAGTTTGAGCGAGGGACCACAATTTACCTAC/ GGAACTTGGACCTCTCACACTC |
|  | Tov278 | TCTA | 21 22 24 25 26 28 32 20 | GCCGACTTCGAGTTTGAGGGTGGAAGACAAATGTCACAGA/ AGTTGTTTGGCAATTAGCCTGT |
| G20 | Tov2 | AAGA | 12 13 14 16 18 19 15 | AGCAAAATAGGCTGTCCCCCACACAGCTAACATGGCTAAA/ TGAGCTGCTGTTGTTTGAAAGT |
|  | Tov214 | GCT | 12 16 17 18 19 9 | AGCAAAATAGGCTGTCCCCCTTTAGCTCAGCACATCCTCT/ GCGCTGTTTGTTTGATGTCTAC |
|  | Tov464 | ATATC | 12 13 14 15 16 10 | AGCAAAATAGGCTGTCCCCTCCACTAACTGACCAGCTCCT/ AATCCTCCACTGTCTCACTGCT |
|  | Tov218 | TCTA | 17 18 19 20 21 24 22 | AGCAAAATAGGCTGTCCCATGGCAAGCTCAATGAGATGTA/ CTTAAATGAATGGCCTTTGCTC |
|  | Tov553 | GGTT | 6 7 8 9 10 12 13 11 | AGCAAAATAGGCTGTCCCACATTTCGACACATCTGAATGG/ CATGCGTGTGATTATGTGTCTG |
|  | Tov141 | AGAT | 15 16 17 18 19 21 22 23 | GCCGACTTCGAGTTTGAGTTTCCTCTCTATTTCTGCGCTC/ TGATTATGCTGCAAACCAATTC |
|  | Tov314 | TCTA | 18 21 22 23 25 26 20 | GCCGACTTCGAGTTTGAGAGAACATCAACTTTGTCCGCTT/ GTAACTTCTCGGCATTGGAATC |
|  | Tov60 | AAGA | 12 14 15 17 18 19 16 | GCCGACTTCGAGTTTGAGGTTGCTGATGATGTTGTGACCT/ CTCCATCACTCAGAGTAGCCCT |
|  | Tov448 | TATC | 16 17 18 20 21 22 19 | GCCGACTTCGAGTTTGAGGGATCAACTGGTTGAGTTCTCC/ GCATTTATGTGACAGCTGGAGT |
| G25 | Tov239 | ATA | 15 17 18 20 21 23 24 26 14 | TGTAAAACGACGGCCAGTCCAGTAACAATCAATGAAGCCA/ ACAATTCACAACTGGACCACTG |
|  | Tov519 | GTGC | 6 10 11 12 13 15 14 | TGTAAAACGACGGCCAGTTTCATGTGATAAACGCAAGGAC/ GTAGCAAATGTGGTGACAGGAA |
|  | Tov50 | GAGAA | 9 10 11 12 14 15 8 | TGTAAAACGACGGCCAGTGGGAGAGGTAGCGATACAAAGA/ TTGACAAGACTGAACGCTGTTT |
|  | Tov85 | AAT | 11 14 15 16 17 20 10 | TGTAAAACGACGGCCAGTTAGTGATTACACCGTCACTGCC/ TGACTGTAGAAAGGCCAATGTG |
|  | Tov326 | GAAA | 17 18 20 21 22 27 19 | TGTAAAACGACGGCCAGTTCCTCCTCTCACTGTTGTCTCA/ TGGTTCCTGGTGTTAGGAGACT |
|  | Tov564 | AGA | 14 16 17 18 19 13 | TTGAGAGGATCGCATCCAAGATGTGCTGCACTACACCAAC/ AAGTTAACAGGACCTGAGCGAG |
|  | Tov243 | TTA | 19 20 21 22 23 30 31 32 16 | TTGAGAGGATCGCATCCAAAGCCACTGTCTGCTGTGTAAA/ GACACAGACTAGAGAGCCAGCA |
|  | Tov567 | AGAT | 16 18 19 20 20.5 22 23 21 | TTGAGAGGATCGCATCCATGCAGGTCTGAGGTCAGTTAAA/ AAGGTCTCACAGTCCACATTCA |
|  | Tov22 | TCAG | 9 10 11 13 14 15 7 | TTGAGAGGATCGCATCCATGGCTGAACAATGGTTGAAATA/ ACAGGACACAGGGCTAATGAAC |
| G36 | Tov368 | CAGA | 12 14 15 19 21 16 | TGTAAAACGACGGCCAGTAACGCTGGAATAAACTAGGCAG/ TGTTTCTGTTTGACTGAATGGG |
|  | Tov178 | TCTG | 5 11 12 15 16 18 19 20 21 23 24 13 | TGTAAAACGACGGCCAGTCTCAGAGGCAGATGTTCAGATG/ AGTGCATCCGATGTATTGTCAG |
|  | Tov82 | GATA | 7 9 10 13 14 11 | TGTAAAACGACGGCCAGTCAGAGATTAAACCAATCAGGGC/ ATTGTTTCAACCATGATCACCA |
|  | Tov533 | GATA | 18 19 20 21 22 14 | TGTAAAACGACGGCCAGTTTCCTGTCTGTTGATGTTGTCC/ CCAATGCAACAAAGCACTTAGA |
|  | Tov530 | AAGA | 13 14 15 16 17 12 | TGTAAAACGACGGCCAGTAACAGCCCAATCAAACTCAACT/ GAAGCCAGATTCAAAGGAAATG |
|  | Tov425 | TAT | 17 18 20 22 23 26 16 | TTGAGAGGATCGCATCCATTGGATAAACACCACACACCAT/ CCTAAATTGTCCATGTGCTGAA |
|  | Tov215 | TAGA | 9 10 11 12 14 15 17 19 8 | TTGAGAGGATCGCATCCATTGCCCAGTTGTAGCAATGTAG/ AAATCCTTTGTTCCCTCTGTCA |
|  | Tov207 | ATTAT | 12 13 16 17 18 19 10 | TTGAGAGGATCGCATCCAACCGTATAGTCGCTAGAGCAGC/ TGTTATTTCACCTACCCTCGCT |
|  | Tov91 | TAA | 12 13 14 16 17 18 20 21 22 10 | TTGAGAGGATCGCATCCAGGGAGTCAGTAGTTTGCTCACC/ ACTGCAGAACAAGTCTGAACCA |
| G42 | Tov18 | TTTAA | 10 12 14 15 16 6 | TGTAAAACGACGGCCAGTCATTGTCCATCTCCCAGGTAAT/ CTACTGATGGACTCATCGCTTG |
|  | Tov357 | TGAG | 9 10 11 13 15 14 | TGTAAAACGACGGCCAGTTGAGACAAGGTCCACTCACTTG/ GGCACTGCTGACTCTCCTCTAT |
|  | Tov80 | ATCT | 9 12 13 14 15 10 | TGTAAAACGACGGCCAGTATGAAAGCTGTATTCTGCCACC/ CCTTGTGTTCCCTTGAGTATGA |
|  | Tov400 | AAT | 7 10 11 12 13 9 | TGTAAAACGACGGCCAGTGTGAGCATAGACACGACTCAGG/ GGATCTTGCAGGGTCACTACTC |
|  | Tov487 | AGAT | 14 15 16 18 19 20 21 25 26 17 | TGTAAAACGACGGCCAGTTTCTTTGTTCCTATTGGCCCTA/ TGCACATACCAGGGTAACAGTC |
|  | Tov105 | AAG | 12 12.3333 12.6667 13 14 9 | TTGAGAGGATCGCATCCAGTTCCGCTTCCTTTAGGAATTT/ GCTCGTTTATGGGAATGAAGTC |
|  | Tov25 | TAGA | 20 23 24 25 27 22 | TTGAGAGGATCGCATCCACCTCATGGTGCTCAACAAATTA/ TGCTTTCTTTCTGAGAGTGCAG |
|  | Tov307 | GAGAA | 8 9 11 12 13 14 6 | TTGAGAGGATCGCATCCAATCTCTCCTCTTTCTGTTTGCG/ GATTTGCTGATTATCCTCCTCG |
|  | Tov92 | AGAT | 15 17 18 19 20 21 22 23 | TTGAGAGGATCGCATCCAGTTTCTGTCCACATGACTCCAC/ CACACCACAGAGCTTGTTAAGG |
| V1 | Tov19037 | GTGC | 10 11 13 14 15 16 12 | TGTAAAACGACGGCCAGTTGGCTCTCAGTGCTATGGTG/ TTGTCTCAGCCCTACAGCCT |
|  | Tov32142 | AACA | 7 10 11 14 12 | TGTAAAACGACGGCCAGTTCCTCCAGTAGGTTTGGTGC/ GAGCTGTCTCTGTGCTGTGC |
|  | Tov25439 | CCTCT | 14 16 19 13 | TGTAAAACGACGGCCAGTAGGTGGCTCTAATCCAGGGT/ TCACTACAGGGACCCACTCC |
|  | Tov26724 | TTCT | 14 16 17 12 | TGTAAAACGACGGCCAGTGGTCAGAGGTCAGGGTGTGT/ TGGCTGAAACTCAACTGTGC |
|  | Tov6129 | AATTG | 16.5 18.5 19.5 6 | TGTAAAACGACGGCCAGTCTGTTGGAGGCTTCTTCCTG/ GGACAAAGGACACAGTCGGT |
|  | Tov34412 | CACA | 28 29 30 | TGTAAAACGACGGCCAGTCAGGCCACTGTGGTTAGGAC/ TTGGAGGATGGTCGTAGAGG |
|  | Tov32776 | AAG | 18 19 20 16 | TTGAGAGGATCGCATCCATGATCTGACTCCCAGCAGTG/ GAGACCTTCCATACGTCCGA |
|  | Tov16774 | AGAGG | 8 12 14 11 | TTGAGAGGATCGCATCCAAACGGAGGGAGGTCAAGTCT/ CCCAGCTCTGATAGCACACA |
|  | Tov15555 | ATGA | 9 12 8 | TTGAGAGGATCGCATCCATGGTGTGTTTGCAGGTCAGT/ CCTGCACCGTATCACTTCCT |
|  | Tov4695 | GATA | 14 16 17 18 19 20 12 | TTGAGAGGATCGCATCCATCCAGCAGCTCTAGGTCCTC/ TTCAAAGGTTGTCCCTCGTC |
