# Supplementary material for: MultiplexSSR: A pipeline for developing multiplex SSR‐PCR assays from resequencing data
Source: Ecol Evol. 2020 Mar 4;10(6):3055–67. doi: 10.1002/ece3.6121 (PMC7083706; doi:10.1002/ece3.6121)
Supplement: Supplementary file 7 [file ECE3-10-3055-s007.doc]

SupTab 5. The universal primer

| Locus | Primer (from 5’ to 3’) | Fluorescein | Color |
| --- | --- | --- | --- |
| RV3 | AGCAAAATAGGCTGTCCC | ROX | Red |
| M13 | TGTAAAACGACGGCCAGT | FAM | Blue |
| PQE-F | TTGAGAGGATCGCATCCA | HEX | Green |
| pVP16 | GCCGACTTCGAGTTTGAG | TAMRA | Yellow |
