# Supplementary material for: MultiplexSSR: A pipeline for developing multiplex SSR‐PCR assays from resequencing data
Source: Ecol Evol. 2020 Mar 4;10(6):3055–67. doi: 10.1002/ece3.6121 (PMC7083706; doi:10.1002/ece3.6121)
Supplement: Supplementary file 8 [file ECE3-10-3055-s008.doc]

SupTab 6. Summary statistics for 13 microsatellite loci in the mass cross population (PM2018).

|  | Tov32142 | Tov25439 | Tov26724 | Tov6129 | Tov32776 | Tov16774 | Tov15555 | Tov4695 | Tov368 | Tov82 | Tov533 | Tov530 | Tov215 |
| --- | --- | --- | --- | --- | --- | --- | --- | --- | --- | --- | --- | --- | --- |
| N | 1681 | 1686 | 1652 | 1710 | 1688 | 1714 | 1716 | 1705 | 1653 | 1658 | 1518 | 1646 | 1610 |
| Na | 4 | 3 | 5 | 2 | 9 | 5 | 4 | 7 | 11 | 7 | 10 | 6 | 10 |
| Ne | 1.514 | 1.444 | 3.744 | 1.599 | 4.392 | 2.037 | 1.820 | 4.036 | 3.389 | 4.145 | 4.566 | 2.925 | 3.887 |
| I | 0.628 | 0.490 | 1.353 | 0.562 | 1.571 | 0.779 | 0.747 | 1.453 | 1.496 | 1.534 | 1.594 | 1.234 | 1.440 |
| Ho | 0.412 | 0.378 | 0.984 | 0.499 | 0.972 | 0.526 | 0.575 | 0.940 | 0.800 | 0.888 | 0.995 | 0.743 | 0.880 |
| He | 0.340 | 0.307 | 0.733 | 0.375 | 0.772 | 0.509 | 0.451 | 0.752 | 0.705 | 0.759 | 0.781 | 0.658 | 0.743 |
| F | -0.212 | -0.229 | -0.343 | -0.333 | -0.259 | -0.032 | -0.275 | -0.250 | -0.135 | -0.170 | -0.274 | -0.129 | -0.185 |

N, number of individuals in the sample; Aa, allelic number; Ne, number of effective alleles; I, Shannon's Information Index; Ho, observed heterozygosity; He, expected heterozygosity; F, fixation index = (He − Ho) / He = 1 − (Ho / He)
